# Role of Mass Transport in the Deposition, Growth, and Transformation of Calcium Carbonate on Surfaces at High Supersaturation

**DOI:** 10.1021/acs.cgd.1c01505

**Published:** 2022-06-27

**Authors:** Ian J. McPherson, Massimo Peruffo, Patrick R. Unwin

**Affiliations:** Department of Chemistry, University of Warwick, Gibbet Hill Road, Coventry, CV4 7AL, U.K.

## Abstract

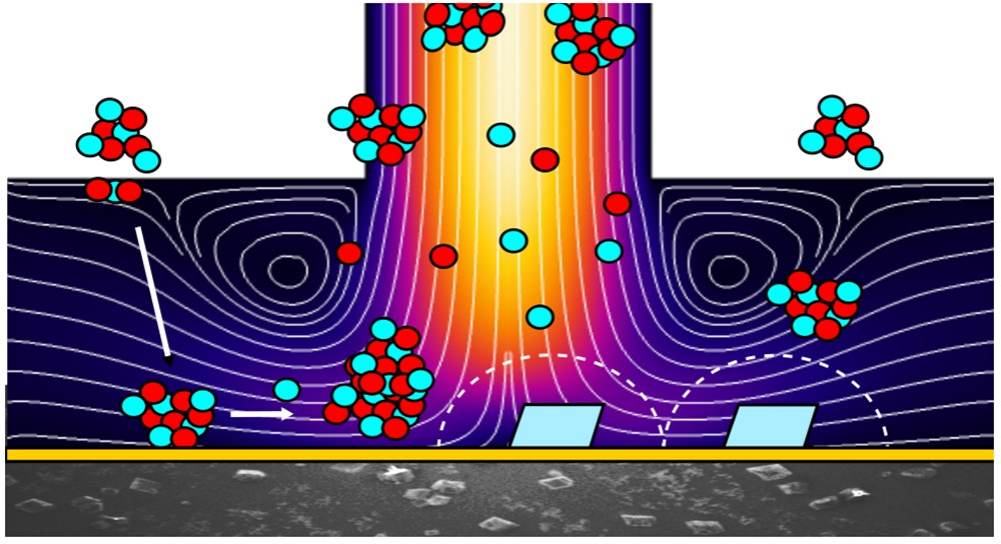

We demonstrate how combined in-situ measurements and finite element method modeling can provide new insight into the relative contribution of mass transport to the growth of calcium carbonate on two model surfaces, glass and gold, under high-supersaturation conditions relevant to surface scaling. An impinging jet-radial flow system is used to create a high-supersaturated solution at the inlet of different cells: an optical microscope cell presenting a glass surface for deposition and quartz crystal microbalance (QCM) and in-situ IR spectroscopy cells, both presenting a gold surface. The approach described is quantitative due to the well-defined mass transport, and both time-lapse optical microscopy images and QCM data are analyzed to provide information on the growth kinetics of the calcite crystals. Initially, amorphous calcium carbonate (ACC), formed in solution, dominates the deposition process. At longer times, the growth of calcite is more significant and, on glass, is observed to consume ACC from the surface, leading to surface regions depleted of ACC developing around calcite microcrystals. On Au, the mass increase becomes linear with time in this region. Taken together, these microscopic and macroscopic measurements demonstrate that calcite growth has a significant component of mass transport control at high supersaturation. Finite element method (FEM) simulations of mass-transport-limited crystal growth support the strong mass transport contribution to the growth kinetics and further suggest that the observed growth must be sustained by more than just the Ca^2+^ and CO_3_^2–^ in solution, with dissolution/direct attachment of ACC and/or ion pairs also contributing to the growth process.

## Abstract 2

In-situ observation of calcite growth from supersaturated solution reveals growth rates consistent with mass transport controlled kinetics.

## 1 Introduction

Calcium carbonate deposition is of primary importance in the fields of chemistry (including industrial and commercial applications),^1^ geochemistry,^2^ and the life sciences,^3^ and much effort has been made to understand its nucleation^4,5^ and subsequent growth kinetics. It is now known that the initial nucleation and orientation of calcium carbonate crystals is controlled to some degree by lattice match and stereochemistry,^6^ hydrophobicity,^7^ surface energy,^8^ and surface charge,^9^ although deconvoluting their relative importance remains a challenge. The subsequent growth of crystals is generally assumed to be controlled by surface kinetics (as opposed to diffusion), based on early seeded growth studies^10−12^ (although mass transport is acknowledged to play a role in growth in porous media).^13^ Later work using atomic force microscopy (AFM) reported the surface kinetics of calcite crystal growth, emphasizing the difficulty in formulating detailed kinetic laws for macroscale behavior without knowledge of the underlying microstructure.^14^ However, more recent microfluidics experiments on single calcite microparticles show much higher growth rates than either AFM or seeded experiments at equivalent supersaturation, a discrepancy attributed in part to differences in mass transport between the techniques.^15^ Subsequent re-evaluation of surface concentrations in AFM, derived from reported critical step lengths, with supporting finite element method (FEM) simulations, highlights that mass transport is an important factor, and there is a strong concentration gradient (boundary layer) for calcite growth under some conditions.^16^

Often, transport effects are assumed to be absent based on the apparent independence of crystal growth on rates of forced convection;^10,14^ however, the concentration boundary layer (CBL) thickness is not a linear function of convection rate, and a limiting boundary layer, and hence mass transport limitation, can persist even under high convection rates.^16,17^ A relatively straightforward, yet underused, method to elucidate surface kinetic control versus diffusion control is to examine growth rate as a function of time. Pure surface control depends on the crystal surface area, and as such the mass growth rate (as opposed to step or edge velocity) in this scenario will increase as a cubic function of time, *t*^3^. Deviations from this behavior, especially a constant mass growth rate, indicates that other factors must be contributing to the rate. Indeed, the mass transport regime will change during the significant growth seen at high supersaturations, as diffusion fields will be localized to widely spaced (small) crystals but then overlap so that bulk-convection-diffusion will then control the transport rate. Many studies of calcite deposition make use of flow-through cells to maintain constant bulk conditions,^13,15,18−22^ and while the effects of flow rate are sometimes considered (usually to find the point of flow rate independence), the time dependence of growth is rarely assessed. Exceptionally, studies using the quartz crystal microbalance (QCM)^22−25^ directly measure deposited mass as a function of time, although little is made of the time dependence in kinetic analyses.

Here, we analyze the deposition and growth of calcium carbonate at high supersaturation as a function of time, under the well-defined mass transport of an impinging jet-radial flow cell, with three different methods: optical microscopy, infrared spectroscopy, and quartz crystal microgravimetry. We then compare the measured growth rates with numerical simulations of mass transport to understand its contribution under various scenarios. Significantly, we show that even under conditions of efficient solution mixing and delivery, and with a reservoir of amorphous precursors on the surface, calcite growth rates can show considerable mass transport control. This behavior has important implications: mass transport phenomena and concentration gradients will be much more widespread than previously recognized. The approach we describe is widely applicable and should be of use to further understand the relative importance of mass transport in the deposition and growth of numerous other mineral systems.^4,26^

## 2 Materials and Methods

### 2.1 Chemicals

Solutions with total calcium ion concentration, [Ca^2+^]_T_ = 20 mM, and total carbonate (all species considered), [CO_3_^2–^]_T_ = 10 mM, were prepared by dissolving, respectively, CaCl_2_·2H_2_O (Sigma ultra, 99%) and NaHCO_3_ (BDH, AnalR 99.5%) salts in Milli-Q grade water (Millipore Corp., 18.2 MΩ cm at 25 °C). NaOH (1 M) (Fisher Scientific, 97%) was added to the carbonate solution to increase the pH to 10.50 ± 0.05. The pH (10.50) and concentrations of carbonate and calcium were chosen to obtain CO_3_^2–^ as the limiting species for deposition (i.e., Ca^2+^ in considerable excess). The solutions were prepared and used as quickly as possible to minimize the exchange of CO_2_ with the atmosphere and satisfy the assumption of a closed system.

### 2.2 Supersaturation Calculations

The nominal free ion concentrations of the as-mixed, unreacted, solutions were calculated using PHREEQC Interactive (v 3.4)^27^ with relevant values shown in Table 1. The simulations considered a closed system at 22 °C and neglected the contribution of atmospheric CO_2_, due to its low concentration. Under these assumptions, the pH of the solutions before adjustment and the amount of NaOH added to reach pH 10.50 agreed well with the experimental values, providing confidence in the model used.

**Table 1:** Nominal Concentration of Ca^2+^ and CO_3_^2–^ Containing Species in the Mixed Solution

species	concentration (mol dm^–3^)
Ca^2+^	7.233 × 10^–3^
CO_3_^2–^	8.325 × 10^–4^
CaCO_3_^0^	2.698 × 10^–3^
CaHCO_3_^+^	6.259 × 10^–5^
NaCO_3_^–^	6.132 × 10^–5^
NaHCO_3_^0^	4.771 × 10^–6^
CaOH^+^	6.637 × 10^–6^

The supersaturation, *S*, with respect to a solid CaCO_3_ phase with the solubility product, *K*_SP_, is defined as
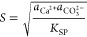
1where *a*_i_ is the activity of species *i* and is calculated from the concentrations using the Davies equation with an ionic strength of 3.12 × 10^–2^ mol dm^–3^. Supersaturation with respect to a range of CaCO_3_ polymorphs can then be calculated (Table 2).

**Table 2:** Solubility Product and Supersaturation with Respect to a Range of CaCO_3_ Polymorphs

phase	*K*_SP_ (mol^2^ dm^–6^)	*S*
calcite^28^	3.47 × 10^–9^	22
aragonite^28^	4.79 × 10^–9^	19
vaterite^28^	1.3 × 10^–8^	12
amorphous CaCO_3_ (ACC)^29^	3.8 × 10^–8^	7

### 2.3 Flow Measurements

**Figure 1:**
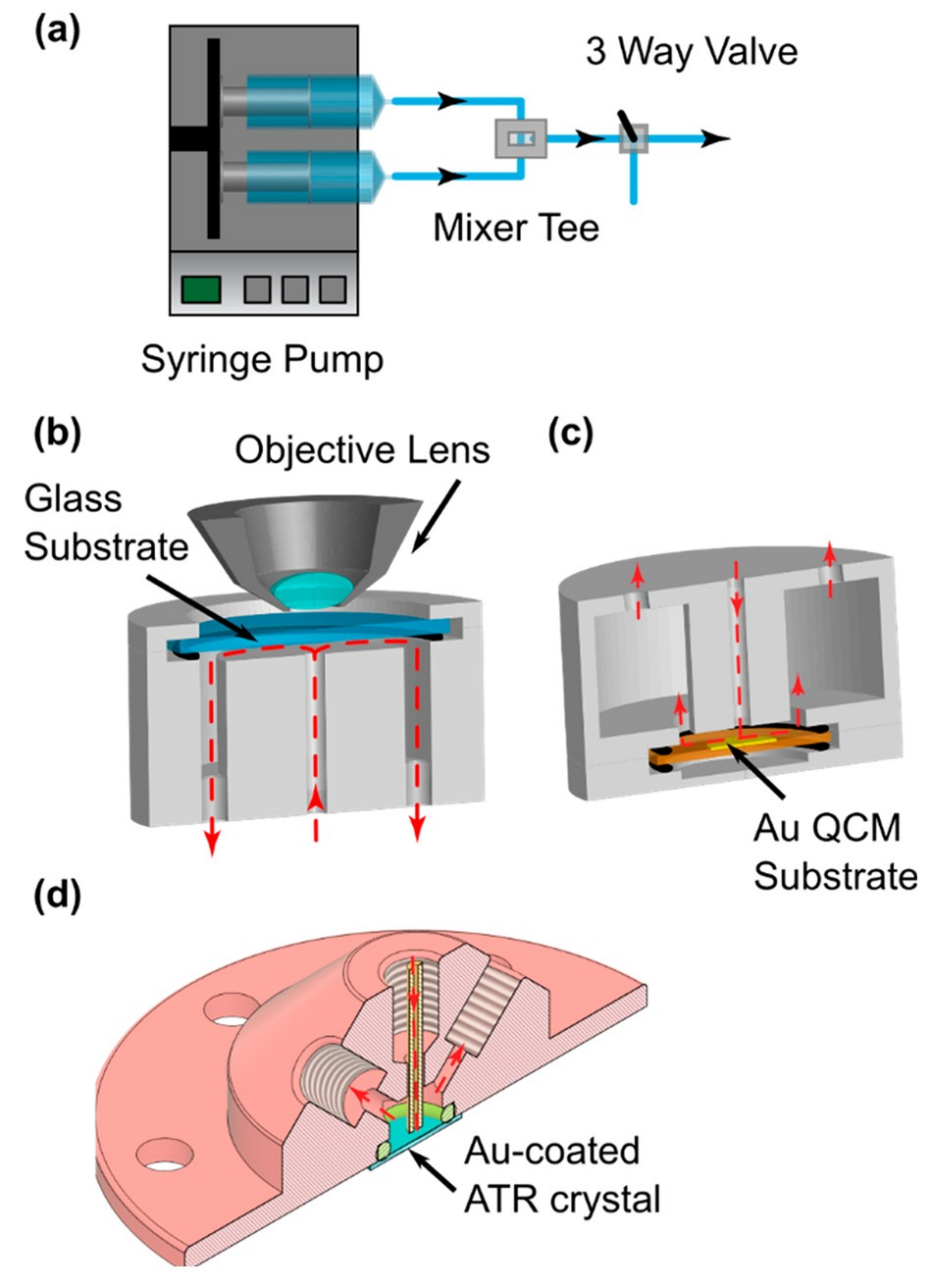
Schematic of the solution delivery system and flow cells used. (a) Solution path, (b) microscopy flow cell, (c) QCM flow cell, and (d) ATR-IR flow cell. Red lines indicate the solution direction.

Solutions were flowed through the system using a dual drive syringe pump system (*K*_d_ Scientific mod. 200, B–D plastic syringes, 60 mL) and mixed in a home-built T-mixer (Figure 1a), with all wetted parts after the mixer constructed from PTFE to avoid heterogeneous nucleation of CaCO_3_. The system provided a constant flow rate (tested in the range of 0.5–1.5 mL min^–1^ for each channel) over long time periods with an error of 0.5% measured, before and after the mixer. At a total flow rate of 2 mL min^–1^ (i.e., 1 mL min^–1^ for each channel), as used herein, the time for the solution to reach the reaction cell of interest was measured as ∼22 s, and the mass transport coefficient, *k*_T_, found from FEM simulations (*vide infra*) was 1.59 × 10^–5^ m s^–1^ (averaged across the whole surface) or 4.82 × 10^–5^ m s^–1^ (directly underneath the nozzle). This compares to a mass transport rate for a diffusionally isolated microcrystal, *k*_T_ ≈ *D*/*r*, where *D* ≈ 10^–5^ cm^2^ s^–1^ is the diffusion coefficient of carbonate (*vide infra* for the precise value), and *r* (∼5 μm) is the typical crystal dimension at short times, ca. 2 × 10^–4^ m s^–1^.

The optical and QCM flow cells were characterized predominantly by impinging jet hydrodynamics (under the nozzle) with a transition to radial flow to the outlet and were designed to have the same geometry, with a 1.5 mm thickness, to obtain similar mass transport and deposition conditions. Between experiments, the solution delivery system was carefully rinsed by flowing through sequentially 120 mL of water, 120 mL HCl solution (pH 3), and 120 mL of water. The PTFE flow cells were kept in acidic solution for 30 min and thoroughly rinsed with water, before and after each experiment.

For in-situ optical microscopy investigations, where the foreign substrate was glass, the PTFE flow cell (Figure 1b) was designed to sit on an upright microscope stage (Leica DM4000 M with a CCD camera DFC490), and the focus centered on the glass surface in a position aligned with the nozzle outlet. To make measurements, the cell was first filled with water, then the supersaturated solution was flowed at 2 mL min^–1^ for a preset time, and images were recorded typically every 5 s (a total of 200 images taking 1000 s) using dark field microscopy to enhance the observation of crystal shape. Images shown in the Supporting Movie (duration 1000 s) were processed in ImageJ using a bandpass filter (2 pixels < passband <50 pixels), and the color table of the darkfield images was inverted for clarity. At the end of an experiment, the cell was filled with air, and the glass substrate was quickly taken off and rinsed with acetone and dried with nitrogen for further analysis. The glass substrates were cut in two pieces through the center and used, respectively, for micro-Raman spectroscopy and field emission scanning electron microscopy (FE-SEM, Zeiss SUPRA 55 VP FE-SEM) imaging (after gold coating).

For QCM studies, using a CHI400 EQCM (CH Instruments), the PTFE cell (Figure 1c) accommodated a gold-coated quartz QCM crystal in the center underneath the nozzle outlet. Fresh gold-coated quartz crystals were cleaned sequentially before use using water, chloroform, and acetone, and then blown dry with nitrogen. Experiments followed a similar procedure to that for in-situ optical microscopy, but CaCO_3_ deposition was monitored via the quartz crystal resonant frequency recorded as a function of time and then converted to mass deposited on the crystal surface using the Sauerbrey equation:^30^
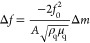
2where Δ*f* is the variation of frequency, Δ*m* is the change in the mass deposited, *f*_0_ is the resonant frequency of the crystal (∼7.995 MHz), *A* is the area of the gold disk coated onto the quartz crystal (0.205 cm^2^), μ_q_ is the shear modulus of the quartz (2.947 × 10^11^ g cm^−1^ s^−1^), and ρ_q_ is the density of the quartz (2.648 g cm^–3^). After experiments, the cell was emptied by flowing in air, and the QCM probe was removed and used for FE-SEM imaging (after gold coating).

The ATR-IR cell (Figure 1d) was 3D printed in clear methacrylate resin (Form Labs, USA) and set on a Si optic (Universal ATR crystal, IRUBIS GmbH), coated with 3.5 nm Ti and 20 nm Au, and mounted on a Veemax III specular reflection accessory in a Vertex 70v FTIR spectrometer (Bruker) with an MCT detector. For in-situ ATR-IR measurements, a spectral collection was started with the cell dry before the solutions were flowed into a mixer tee connected directly to the flow cell at a rate of 1 mL min^–1^. Each spectrum consisted of 32 scans coadded to give a time resolution of 15 s. The final data set was generated by averaging four spectra and using the first spectrum as a background. Peak fitting was carried out in OriginPro using Gaussian peak shapes.

### 2.4 Finite Element Method Modeling

Mass transport in the flow cells was modeled in COMSOL (v5.6) using the Transport of Dilute Species and Laminar Flow modules. Where used, concentration boundary conditions were calculated in PHREEQC.^27^

## 3 Results and Discussion

### 3.1 Visualization and Analysis of CaCO_3_ Deposition by in-Situ Optical Microscopy

**Figure 2:**
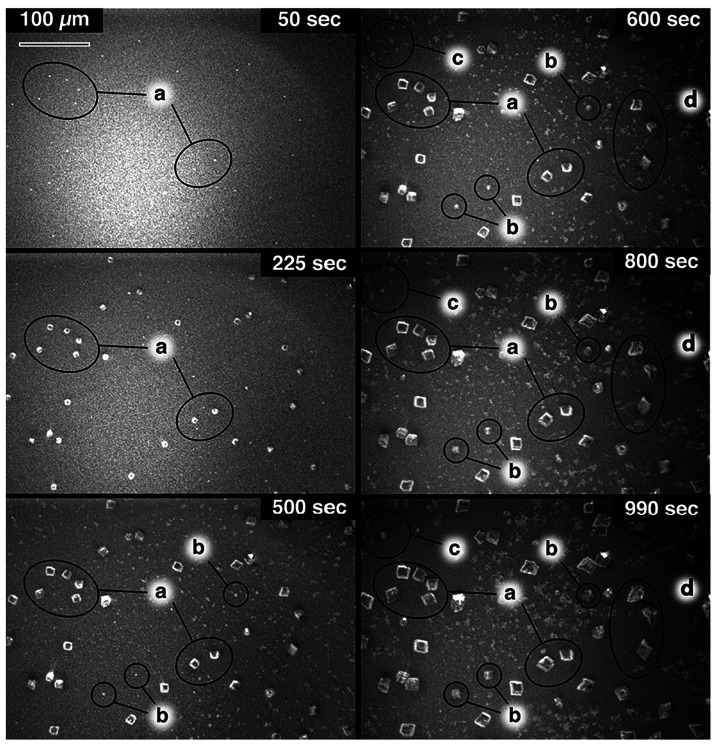
Time-lapse images recorded with the in-situ optical microscopy set up at 50, 225, 500, 600, 800, and 990 s. Regions labeled “a” highlight the initial heterogeneous nucleation of calcite. Regions labeled “b” and “c” indicate phase change nucleation, and regions marked “d” show depletion. The Supporting Movie, from which these frames are taken, is supplied separately.

First, dark field imaging was used to follow CaCO_3_ deposition onto glass slides. Solutions of 20 mM CaCl_2_ and 10 mM NaHCO_3_ were mixed and flowed over a microscope slide, while time-lapse images were recorded (Figure 2). The images (combined into a Supporting Movie) show the rapid appearance of rhombohedral crystals (consistent with calcite, as confirmed by micro-Raman, Supporting Information, section S2) as well as smaller flocks of material over the glass surface, consistent with either ACC or vaterite nanoparticles. At the high supersaturation used, ACC is likely to form instantly via spinodal decomposition,^31,32^ with the flow then transporting the ACC to the surface where it may attach and transform into vaterite and/or calcite. At later times (after ∼800 s), the ACC nearby the calcite crystals starts to dissolve, for example, in the regions labeled “a” and “d” in Figure 2. This process is initially quite subtle, but as the calcite crystal size increases, surface depletion of ACC becomes more significant and is clearly visible in the time-lapse Supporting Movie. Evidently, the growing calcite crystals consume material at such a rate that, at the glass/solution interface, the solution becomes undersaturated with respect to ACC, driving its dissolution. Enhanced growth of faces pointing into the flow field is also seen, particularly at longer times where the crystal is larger, manifesting as distortions to the otherwise regular rhombohedra. This indicates that convection augments mass transport and that its contribution generally increases with distance above the macroscopic substrate, in line with the velocity profile for laminar flow over a surface (although the crystals will, of course, distort the flow).

The fact that ACC dissolution at the surface occurs mainly in regions where there are also calcite crystals indicates there is a strong concentration gradient around the calcite crystals in those regions, with the calcite crystals acting as a sink for calcium carbonate. It is even more striking that this occurs despite the continued flow of solution from the bulk, which will be at equilibrium, or even supersaturated, with respect to ACC (as confirmed by the continued growth of ACC deposits in regions far from calcite). Thus, under the prevailing conditions, calcite growth kinetics is sufficiently fast to lead to depletion of calcium and carbonate from solution at the crystal surface and therefore introduces a strong component of mass transport control into the ACC-calcite transformation and growth process. To address this aspect more quantitatively, the growth rates of individual calcite crystals were analyzed.

Heterogeneous nucleation and growth of calcite normally occur on the more stable (10.4) surface,^33^ and the calcite crystals formed on the glass surface show this characteristic, with 5 (10.4) planes exposed to the solution. The surface coverage of calcite crystals formed in the initial nucleation/growth process was 2.3 ± 0.2 × 10^4^ cm^–2^. The second nucleation stage produced only a few extra crystals, 6 ± 2 × 10^3^ cm^–2^, giving a total of 2.9 ± 0.3 × 10^4^ cm^–2^.

**Figure 3:**
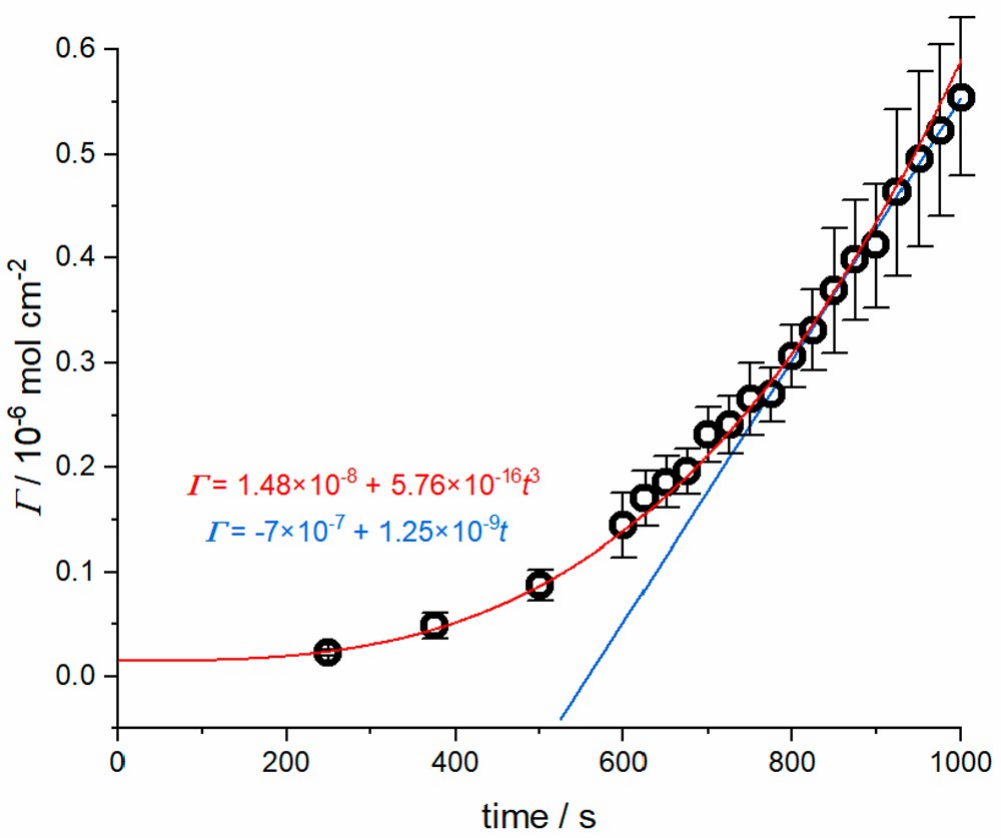
Estimated crystal size over time evaluated from in-situ optical images.

To estimate the growth rate, three crystals with a well-defined rhombohedral shape growing from the (10.4) surface were chosen, and their dimensions were evaluated as a function of time. The measurements were made for times greater than 250 s when the crystals were sufficiently large to measure the dimensions with reasonable precision. The volume of the growing crystals was calculated from the relation *V* = *d*_maj_^3^ × 0.0893 (where *d*_maj_ is the major diagonal; see SI section S1, Figure S1). Converting the volume into moles and multiplying by the observed crystal density on the substrate give the approximate amount of CaCO_3_ deposited over time, Γ_CaCO_3__ (Figure 3).^34^ The deposition rate (flux) gradually increases over time, which is broadly consistent with the *t*^3^ dependence expected for surface kinetics controlled growth with a constant flux (red line, Figure 3). In this regime, mass transport rates are high to small isolated microcrystals, and so surface kinetics may be expected (*vide supra*). The deposition rate reaches a limiting value in the final ca. 300 s, described by a linear flux, 1.25 ± 0.03 × 10^–9^ mol cm^–2^ s^–1^ (blue line, Figure 3, Table 3). In this regime, there is increasing diffusional interaction between neighboring crystals, and mass transport control is well described by macroscopic convection-diffusion, as confirmed by simulations of the expected flux to the substrate using FEM (*vide infra*).

**Table 3:** Measurements from in-Situ Growth Experiments

substrate	crystal density (cm^–2^)	limiting growth rate/crystal (10^–14^ mol s^–1^)	limiting flux of CaCO_3_ to substrate surface (10^–9^ mol cm^–2^ s^–1^)
glass	2.9 ± 0.3 × 10^4^	5.5 ± 0.2	1.25 ± 0.03
gold	2.3 ± 0.2 × 10^5^ (center)		3.5 ± 0.1
	1.9 ± 0.2 × 10^5^ (edge)		

### 3.2 ACC-Calcite Transformation

**Figure 4:**
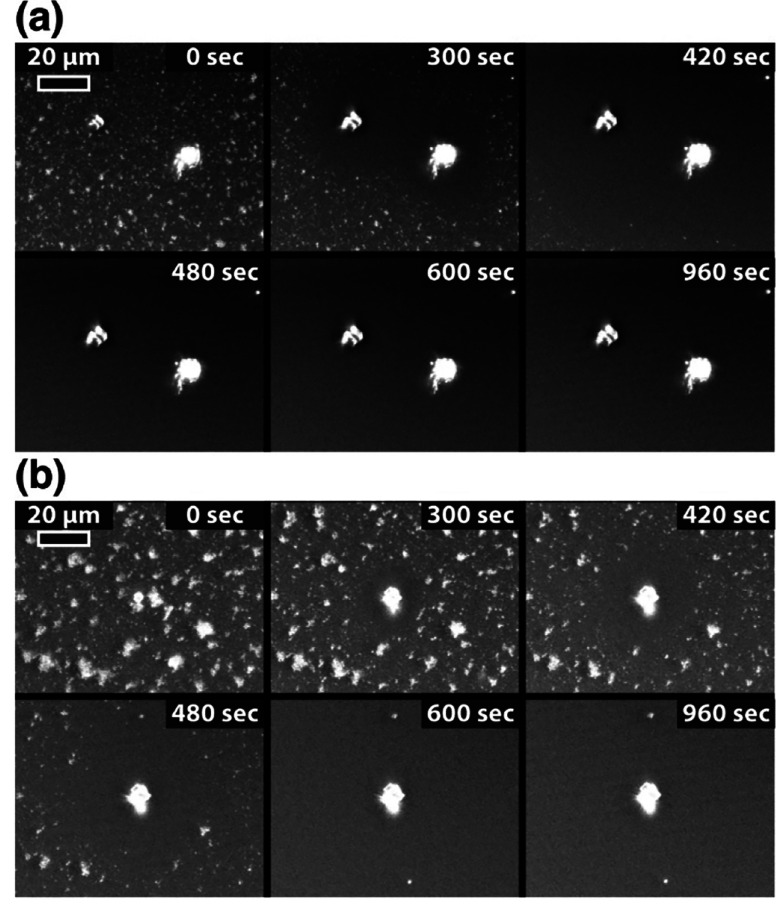
Time-lapse pictures recorded with the in-situ optical microscopy set up. ACC dissolution process in the presence of overlap of the diffusion field for two closely spaced growing calcite crystals (a) and for a single growing calcite crystal (b).

To track the ACC-calcite transformation process in more detail, both calcite and ACC were deposited on the glass substrate following the previous protocol, but after 725 s of deposition the flow was stopped, and time-lapse images were recorded (Figure 4). Using this procedure, the ACC dissolution process is accelerated compared to the previous experiment in which fresh material was continuously delivered to the surface by flow. Without fresh solution flow, calcium and carbonate taken up by the growing calcite crystals are depleted rapidly. When an undersaturated solution with respect to ACC is attained, dissolution of ACC commences, initially close to the calcite crystals then gradually across the whole surface. The time-lapse images show that areas of the surface of circular shape become clear of ACC, with a calcite crystal at the center. This pattern is typical of a diffusive process to a microscopic object.^35^

### 3.3 CaCO_3_ Deposition on Au

**Figure 5:**
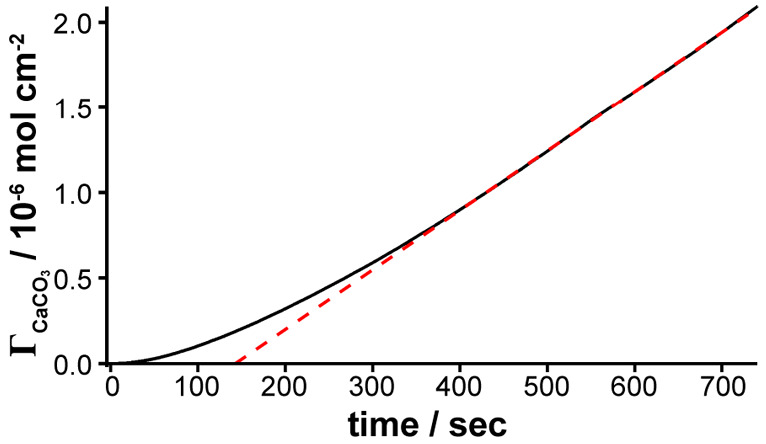
Deposition of calcium carbonate, using the QCM flow cell set up, as a function of time. The red dashed line shows the limiting behavior for a constant flux that characterizes the deposition process at longer times.

The CaCO_3_ deposition process was also investigated on a Au-coated QCM chip. The same procedure was used to mix CaCl_2_ and NaHCO_3_ and direct the resulting solution toward the surface. The change in frequency of the QCM chip over time was converted into adsorbed mass using the Sauerbrey equattion (eq 2), which could then be converted into the amount of CaCO_3_ deposited, Γ_CaCO_3__ (Figure 5). As with the optical measurements on glass, at first (*t* < 100 s) the overall flux to the surface is rather low but increases with time, and at longer times a constant flux is attained (indicated by the dashed red line). This situation was reached after ∼400 s, and the gradient thereafter (3.5 ± 0.1 × 10^–9^ mol cm^–2^ s^–1^) is the overall flux to the QCM probe surface, similar to the analysis of optical images of CaCO_3_ on the glass surface.

**Figure 6:**
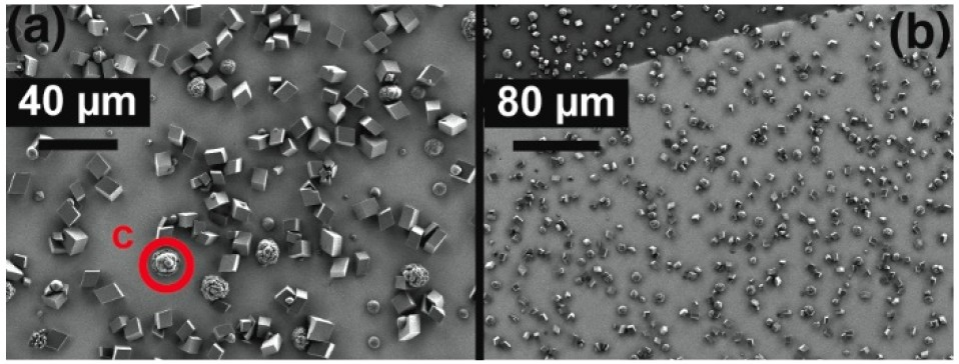
FE-SEM images of the QCM crystal after deposition. The images were taken underneath the nozzle (a) and at the edge of the gold-coated surface (b). Deposits were predominantly rhombohedral calcite, but a few more spherical particles, likely vaterite, were observed (c).

The density of crystals on the Au surface was measured via SEM (Figure 6) and was determined as 2.3 (±0.2) × 10^5^ cm^–2^ at the position directly under the nozzle and 1.9 (±0.2) × 10^5^ cm^–2^ at the substrate edge. The majority of particles were rhombohedral, indicative of calcite, although a few more spherical particles, indicative of vaterite, were observed (Figure 6c). These densities are an order of magnitude larger than on glass and, although not the primary focus of this study, are consistent with the greater persistence of ACC (i.e., slower nucleation of calcite) previously observed on more hydrophilic substrates (including glass above).^7^ The higher density of crystals also explains the weaker time dependence seen on Au compared to glass, as the greater crystal density leads to an earlier transition to bulk mass transport limitation. The small spatial variation in density is a result of the constant supersaturation across the substrate at the early stage of the experiment (where solution will only become slightly depleted in soluble calcium carbonate and ACC) due to the constant flow of solution, which ensures a similar initial driving force is experienced over the whole substrate, resulting in a relatively uniform crystal number density. However, it is important to point out that although the crystal *density* is uniform across the surface, there is a significant variation in size at the end of the experimental run, with microcrystals at the edge being smaller, indicative of more severe mass transport limitations, as a result of the nonuniform flux from an impinging jet. This aspect will be addressed later in simulations of mass transport and concentration profiles.

**Figure 7:**
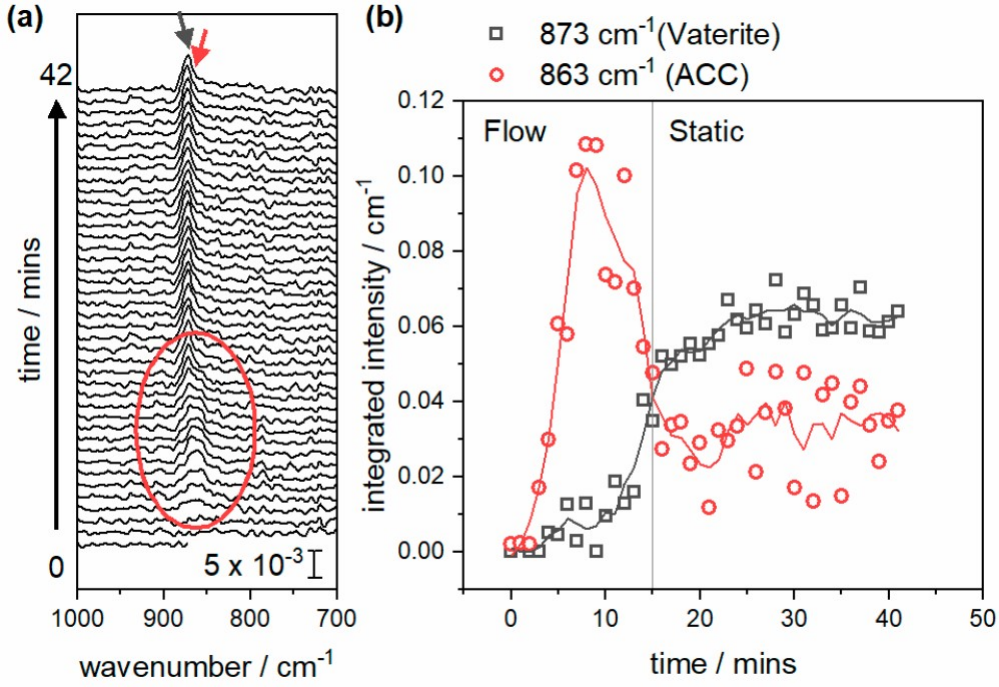
In-situ IR spectra (a) and fitted integrated intensities (b) showing appearance of the ν_2_ mode from ACC (red) and then vaterite (black) over time.

To provide further insight into the speciation near the Au surface during deposition, in-situ ATR-IR spectroscopy using a Au-coated Si optic was performed (Supporting Information, section S3).^36,37^ As before, the calcium and carbonate solutions were mixed and flowed over the Au-coated ATR-IR crystal for 15 min before the flow was stopped while growth continued. Focusing on the ν_2_ mode to discriminate between ACC (863 cm^–1^) and the anhydrous polymorphs (ca. 873 cm^–1^),^37^ the spectra in Figure 7a, reveal the rapid emergence of a broad peak at 863 cm^–1^ (fwhm ca. 25 cm^–1^), which then narrows and shifts to 873 cm^–1^ (fwhm ca. 12 cm^–1^). Peak fitting allows the overlapping contributions to be separated and confirms that the 863 cm^–1^ peak forms fastest but then decays over time and is replaced by 873 cm^–1^ (Figure 7b). This is consistent with the initial formation or attachment of ACC at the surface, followed by transformation to either calcite or vaterite. While the lower wavenumber region, usually the most diagnostic for CaCO_3_ polymorphs, is accessible in situ, the weak nature of the ν_4_ vibration means that no peaks are visible in this region, and so further deconvolution to vaterite or calcite is not possible.

### 3.4 Mass Transport Simulations

To probe the origin of the observed constant flux of calcium carbonate at the substrate at longer times, evident in both the optical and QCM measurements, the mass transport regime of the crystal deposition process was simulated using the finite element method (FEM). This methodology allows realistic modeling of both convection and diffusion in the particular geometry of the flow cell used, in contrast to previous studies which were limited to diffusion in one dimension,^38^ and is facilitated by the flow cell design which establishes well-defined mass transport across the substrate. The CO_3_^2–^ diffusion-limited flux at a calcite microcrystal surrounded by ACC in a flow field flow was estimated considering two inlet solution compositions: (i) negligible reaction on mixing, with speciation determined only by solution phase species and (ii) equilibration with ACC upon mixing.^39^ Mass transport was solved for using the convection-diffusion equation (eq 3), where *c* is the concentration of CO_3_^2–^, and *D* is its diffusion coefficient (0.955 × 10^–5^ cm^2^ s^–1^).^27^ The solution velocity, ***u***, was given by the incompressible Navier–Stokes equation (eq 4), where *p* is the pressure, and ρ and η are the density and dynamic viscosity of water, respectively.
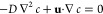
3
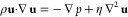
4

**Figure 8:**
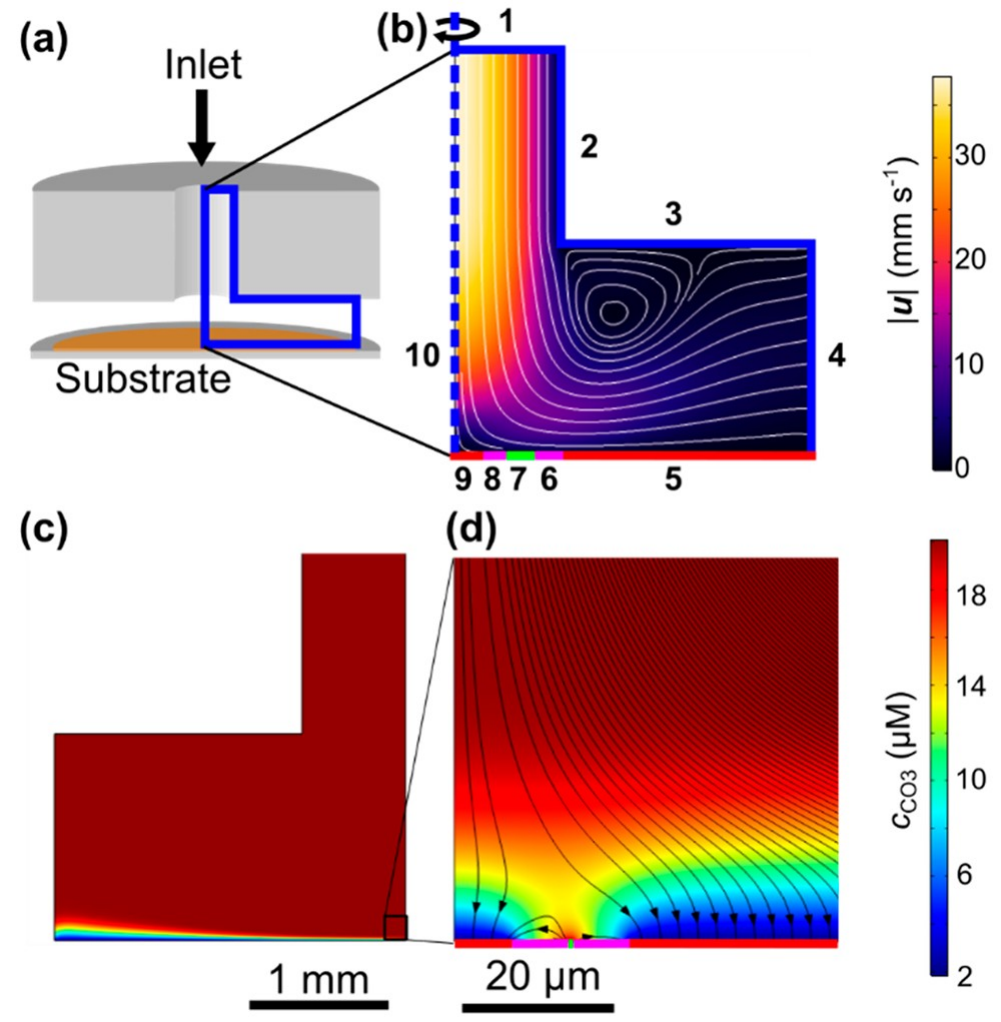
FEM simulation of convection-diffusion in flow cells. *R*_calcite,glass_ = 7.5 μm. Inlet equilibrated with ACC. (a) Relationship between flow cell and FEM domain, (b) enlargement of FEM domain with boundaries labeled, with simulated velocity magnitude overlaid, (c) CO_3_^2–^ concentration, (d) enlarged region of (c) showing calcite and ACC regions. Flux direction marked with streamlines. Note that (c) is reflected in the vertical axis relative to the geometry of (a), (b), and (d).

Boundary conditions for eq 3 were chosen to represent the long-time growth regime (e.g., after ca. 700 s), where calcite is surrounded by ACC on the surface (see above). Since we are seeking a general estimate of the flux and processes that occur under the selected conditions, the calcite crystal (boundary 9) is represented as a cylindrical disk, radius *r*_calcite_, at the center of the substrate, so that the system takes on a simple axisymmetric cylindrical geometry, allowing it to be represented by a 2D geometry with a planar substrate (Figure 8a,b). A constant surface CO_3_^2–^ concentration, *c* = c*_calcite_, given by the concentration of the inlet solution after equilibration with calcite (Table 4), as evaluated in PHREEQC, was applied (corresponding to diffusion-limited growth). Referring to Figure 8a,b, this region was surrounded by a depletion zone equal to *r*_calcite_ and described by a no flux boundary condition (boundary 8). In turn, this was surrounded by a ring of ACC, with a thickness of 0.1*r*_calcite_ (slightly smaller than experimentally observed patches to account for it being continuous, boundary 7) and a constant CO_3_^2–^ concentration, *c* = c*_ACC_, given by the concentration of the inlet solution after equilibration with ACC (Table 1). This was followed by another depletion zone equal to *r*_calcite_ (boundary 6) and finally a semi-infinite layer of calcite (boundary 5, Figure 8a) to simulate the average flux to the remaining surface. Boundary conditions for eq 4 were as follows: fully developed laminar flow consistent with a volume flow rate of 2 mL min^–1^ at boundary 1, zero pressure at boundary 4, no normal velocity at boundary 10, and zero velocity (“no slip”) at all other boundaries.

**Table 4:** CaCO_3_ Phase Surface Boundary Conditions

	CaCO_3_ phase	
species	ACC	calcite
CO_3_^2–^	20.1 μM	1.83 μM
Ca^2+^	6.28 mM	6.18 mM

The experimental flux measured for both the glass and gold substrates lies toward the simulated value for unreacted inlet conditions (Table 5), which yields the maximum mass transport-limited flux at the substrate surface. This analysis thus confirms that mass transport is very important in determining the growth rate of calcite in this system. The simulations also provide an explanation for the variation in particle size seen over the Au surface. Figure 8c shows the steady-state CO_3_^2–^ concentration gradient over the surface when the inlet solution was equilibrated with ACC, as an example, where consumption of material across the substrate leads to increasing depletion at larger radial distances. This depletion leads to an increasing CBL, a lower flux, and hence smaller crystals, at the edges of the substrate. We note that at early times, before significant deposition, depletion will be minimal, and so uniform *nucleation* (and therefore particle density) is observed. The higher crystal density observed on the Au substrate is also consistent with the earlier onset of transport-limited (constant) flux seen on Au (*t*_ons, Au_ ≈ 350 s) compared to glass (*t*_ons,glass_ ≈ 700 s).

**Table 5:** Simulated Limiting CO_3_^2–^ Flux^a^

	inlet condition	
boundary	unreacted	ACC equilibrium
(B9) *R*_calcite,Au_ = 3 μm	8.95 × 10^–9^	5.63 × 10^–10^
(B9) *R*_calcite,glass_ = 7.5 μm	8.46 × 10^–9^	3.18 × 10^–10^
(B5) semi-infinite calcite	1.31 × 10^–9^	2.88 × 10^–11^

a mol cm^–2^ s^–1^.

**Figure 9:**
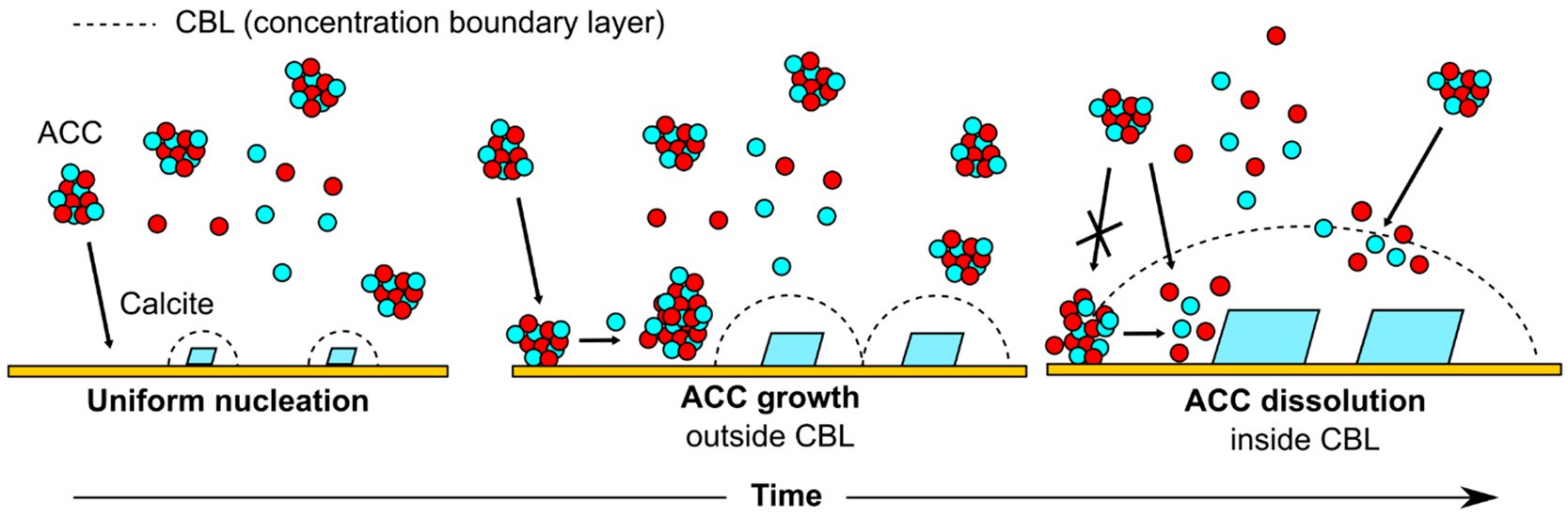
Schematic of calcite growth from ACC-saturated solution at three different stages in the deposition process (see text for details). CBL indicates the concentration boundary layer.

These results show that the deposition of CaCO_3_ on surfaces is a complex, time- and substrate-dependent process, strongly influenced by mass transport. The process consists of three key regimes, illustrated in Figure 9: (1) Formation of ACC in solution by spinodal decomposition,^31^ and uniform nucleation of calcite with little perturbation of the surrounding solution. (2) Growth of calcite and deposition and continued aggregation of ACC. (3) Dissolution of ACC already on the surface, and of incoming aggregates, in the vicinity of calcite, but continued ACC aggregation in other areas. This deposition of ACC and growth of calcite agrees well with previous reports of CaCO_3_ deposition on SiN_*x*_ membranes^39^ and self-assembled monolayers^21^ in quiescent solution. However, the continued supply of solution in the present case enables further conclusions to be drawn. The absence of ACC deposition inside the depletion zone surrounding each calcite crystal, even when deposition occurs away from calcite, implies that ACC dissolution in solution is sufficiently fast that complete conversion back to the ions occurs in the short time between entering the depletion zone and reaching the surface. Alternatively, the measured fluxes are also consistent with the direct attachment of incoming ACC particles to the calcite crystal,^40^ which outcompetes deposition on the substrate. This explains why the measured growth rates are reasonably consistent with the transport-limited flux of unreacted calcium and carbonate ions, as the inlet condition, rather than free ions after equilibration, because the ACC produced upon mixing and delivery (along with ion pairs) is a significant source of Ca^2+^ and CO_3_^2–^, by dissolution or direct attachment to calcite. In general, the dominant regime will be highly dependent on the system size, as well as ion concentrations and diffusivities; an apparent absence of mass transport control is observed in other matrices.^41^

## 4 Conclusions

We have shown how the use of well-defined hydrodynamics, applied with complementary in-situ measurements and mass transport modeling, can be used to understand the deposition and growth of calcium carbonate on foreign surfaces. Deposition occurs via the formation of amorphous calcium carbonate (ACC) in solution, some of which is deposited on the substrate (glass and gold), followed by transformation to vaterite and/or calcite. Flux during deposition tends to constant values at both substrates over time, broadly consistent with mass transport control, showing that crystal growth is strongly limited by the supply of material. In-situ visualization reveals that this material, in part, comes from the initially deposited ACC, as depletion zones develop around the growing crystals. However, FEM modeling of the mass transport suggests that, for a solution in equilibrium with ACC, dissolution of deposited ACC (in combination with free ions in solution) is insufficient to support the observed growth rate and that solution ACC must also contribute to growth, either via rapid dissolution near less soluble calcite crystals or direct attachment.

## Supporting Information Available

A detailed description of the method used to estimate flux from crystal dimensions, a Raman spectrum of the glass substrate after deposition, complete in-situ IR spectra during deposition (PDF)
A movie of the time-lapse data used in Figure 2 (AVI)

## Supplementary Material

cg1c01505_si_001.pdf

cg1c01505_si_002.avi

## References

[ref1] Dalas E.; Koutsoukos P. G. Calcium Carbonate Scale Formation and Prevention in a Flow-through System at Various Temperatures. Desalination 1990, 78 (3), 403–416. 10.1016/0011-9164(90)80060-O.

[ref2] Morse J. W.; Arvidson R. S.; Lüttge A. Calcium Carbonate Formation and Dissolution. Chem. Rev. 2007, 107 (2), 342–381. 10.1021/cr050358j. 17261071

[ref3] Falini G.; Albeck S.; Weiner S.; Addadi L. Control of Aragonite or Calcite Polymorphism by Mollusk Shell Macromolecules. Science 1996, 271 (5245), 67–69. 10.1126/science.271.5245.67.

[ref4] Jun Y.-S.; Kim D.; Neil C. W. Heterogeneous Nucleation and Growth of Nanoparticles at Environmental Interfaces. Acc. Chem. Res. 2016, 49 (9), 1681–1690. 10.1021/acs.accounts.6b00208. 27513685

[ref5] de Yoreo J. J.; Waychunas G. A.; Jun Y.-S.; Fernandez-Martinez A. 7. In Situ Investigations of Carbonate Nucleation on Mineral and Organic Surfaces. In Reviews in Mineralogy & Geochemistry; De Gruyter, 2013; Vol. 77, pp 229–258. 10.1515/9781501508073-009.

[ref6] Aizenberg J.; Black A. J.; Whitesides G. M. Oriented Growth of Calcite Controlled by Self-Assembled Monolayers of Functionalized Alkanethiols Supported on Gold and Silver. J. Am. Chem. Soc. 1999, 121 (18), 4500–4509. 10.1021/ja984254k.

[ref7] Koishi A.; Fernandez-Martinez A.; Van Driessche A. E. S.; Michot L. J.; Pina C. M.; Pimentel C.; Lee B.; Montes-Hernandez G. Surface Wetting Controls Calcium Carbonate Crystallization Kinetics. Chem. Mater. 2019, 31 (9), 3340–3348. 10.1021/acs.chemmater.9b00417.

[ref8] Hamm L. M.; Giuffre A. J.; Han N.; Tao J.; Wang D.; De Yoreo J. J.; Dove P. M. Reconciling Disparate Views of Template-Directed Nucleation through Measurement of Calcite Nucleation Kinetics and Binding Energies. Proc. Natl. Acad. Sci. U. S. A. 2014, 111 (4), 1304–1309. 10.1073/pnas.1312369111. 24434555 PMC3910584

[ref9] Giuffre A. J.; Hamm L. M.; Han N.; De Yoreo J. J.; Dove P. M. Polysaccharide Chemistry Regulates Kinetics of Calcite Nucleation through Competition of Interfacial Energies. Proc. Natl. Acad. Sci. U. S. A. 2013, 110 (23), 9261–9266. 10.1073/pnas.1222162110. 23690577 PMC3677451

[ref10] Nancollas G. H.; Reddy M. M. The Crystallization of Calcium Carbonate. II. Calcite Growth Mechanism. J. Colloid Interface Sci. 1971, 37 (4), 824–830. 10.1016/0021-9797(71)90363-8.

[ref11] Reddy M. M.; Gaillard W. D. Kinetics of Calcium Carbonate (Calcite)-Seeded Crystallization: Influence of Solid/Solution Ratio on the Reaction Rate Constant. J. Colloid Interface Sci. 1981, 80 (1), 171–178. 10.1016/0021-9797(81)90173-9.

[ref12] Reddy M. M.; Plummer L. N.; Busenberg E. Crystal Growth of Calcite from Calcium Bicarbonate Solutions at Constant PCO2 and 25°C: A Test of a Calcite Dissolution Model. Geochim. Cosmochim. Acta 1981, 45 (8), 1281–1289. 10.1016/0016-7037(81)90222-2.

[ref13] Noiriel C.; Steefel C. I.; Yang L.; Bernard D. Effects of Pore-Scale Precipitation on Permeability and Flow. Advances in Water Resources 2016, 95, 125–137. 10.1016/j.advwatres.2015.11.013.

[ref14] Teng H. H.; Dove P. M.; De Yoreo J. J. Kinetics of Calcite Growth: Surface Processes and Relationships to Macroscopic Rate Laws. Geochim. Cosmochim. Acta 2000, 64 (13), 2255–2266. 10.1016/S0016-7037(00)00341-0.

[ref15] Li L.; Sanchez J. R.; Kohler F.; Røyne A.; Dysthe D. K. Microfluidic Control of Nucleation and Growth of CaCO3. Cryst. Growth Des. 2018, 18 (8), 4528–4535. 10.1021/acs.cgd.8b00508.

[ref16] Darkins R.; McPherson I. J.; Ford I. J.; Duffy D. M.; Unwin P. R. Critical Step Length as an Indicator of Surface Supersaturation during Crystal Growth from Solution. Cryst. Growth Des. 2022, 22 (2), 982–986. 10.1021/acs.cgd.1c01249. PMC9097158 35572167

[ref17] Peruffo M.; Mbogoro M. M.; Adobes-Vidal M.; Unwin P. R. Importance of Mass Transport and Spatially Heterogeneous Flux Processes for in Situ Atomic Force Microscopy Measurements of Crystal Growth and Dissolution Kinetics. J. Phys. Chem. C 2016, 120 (22), 12100–12112. 10.1021/acs.jpcc.6b03560.

[ref18] Stockmann G. J.; Wolff-Boenisch D.; Bovet N.; Gislason S. R.; Oelkers E. H. The Role of Silicate Surfaces on Calcite Precipitation Kinetics. Geochim. Cosmochim. Acta 2014, 135, 231–250. 10.1016/j.gca.2014.03.015.

[ref19] Hoeher A.; Mergelsberg S.; Borkiewicz O. J.; Dove P. M.; Michel F. M. A New Method for in Situ Structural Investigations of Nano-Sized Amorphous and Crystalline Materials Using Mixed-Flow Reactors. Acta Cryst. A 2019, 75 (5), 758–765. 10.1107/S2053273319008623. PMC6718202 31475919

[ref20] Devriendt L. S.; Mezger E. M.; Olsen E. K.; Watkins J. M.; Kaczmarek K.; Nehrke G.; de Nooijer L. J.; Reichart G.-J. Sodium Incorporation into Inorganic CaCO3 and Implications for Biogenic Carbonates. Geochim. Cosmochim. Acta 2021, 314, 294–312. 10.1016/j.gca.2021.07.024.

[ref21] Hu Q.; Nielsen M. H.; Freeman C. L.; Hamm L. M.; Tao J.; Lee J. R. I.; Han T. Y. J.; Becker U.; Harding J. H.; Dove P. M.; De Yoreo J. J. The Thermodynamics of Calcite Nucleation at Organic Interfaces: Classical vs. Non-Classical Pathways. Faraday Discuss. 2012, 159, 509–523. 10.1039/c2fd20124k.

[ref22] Cao B.; Stack A. G.; Steefel C. I.; DePaolo D. J.; Lammers L. N.; Hu Y. Investigating Calcite Growth Rates Using a Quartz Crystal Microbalance with Dissipation (QCM-D). Geochim. Cosmochim. Acta 2018, 222, 269–283. 10.1016/j.gca.2017.10.020.

[ref23] Gabrielli C.; Keddam M.; Khalil A.; Maurin G.; Perrot H.; Rosset R.; Zidoune M. Quartz Crystal Microbalance Investigation of Electrochemical Calcium Carbonate Scaling. J. Electrochem. Soc. 1998, 145 (7), 2386 10.1149/1.1838648.

[ref24] Garcia C.; Courbin G.; Ropital F.; Fiaud C. Study of the Scale Inhibition by HEDP in a Channel Flow Cell Using a Quartz Crystal Microbalance. Electrochim. Acta 2001, 46 (7), 973–985. 10.1016/S0013-4686(00)00671-X.

[ref25] Abdel-Aal N.; Satoh K.; Sawada K. Study of the Adhesion Mechanism of CaCO3 Using a Combined Bulk Chemistry/QCM Technique. J. Cryst. Growth 2002, 245 (1–2), 87–100. 10.1016/S0022-0248(02)01657-3.

[ref26] Benning L. G.; Waychunas G. A. Nucleation, Growth, and Aggregation of Mineral Phases: Mechanisms and Kinetic Controls. In Kinetics of Water-Rock Interaction; Brantley S. L., Kubicki J. D., White A. F., Eds.; Springer: New York, NY, 2008; pp 259–333. 10.1007/978-0-387-73563-4_7.

[ref27] Parkhurst D. L.; Appelo C. A. J. Description of Input and Examples for PHREEQC Version 3: A Computer Program for Speciation, Batch-Reaction, One-Dimensional Transport, and Inverse Geochemical Calculations; Techniques and Methods; USGS Numbered Series 6-A43; U.S. Geological Survey: Reston, VA, 2013; p 519.

[ref28] Plummer L. N.; Busenberg E. The Solubilities of Calcite, Aragonite and Vaterite in CO_2_-H_2_O Solutions between 0 and 90°C, and an Evaluation of the Aqueous Model for the System CaCO_3_-CO_2_-H_2_O. Geochim. Cosmochim. Acta 1982, 46 (6), 1011–1040. 10.1016/0016-7037(82)90056-4.

[ref29] Gebauer D.; Völkel A.; Cölfen H. Stable Prenucleation Calcium Carbonate Clusters. Science 2008, 322 (5909), 1819–1822. 10.1126/science.1164271. 19095936

[ref30] Sauerbrey G. Verwendung von Schwingquarzen zur Wägung dünner Schichten und zur Mikrowägung. Z. Physik 1959, 155 (2), 206–222. 10.1007/BF01337937.

[ref31] Zou Z.; Habraken W. J. E. M.; Bertinetti L.; Politi Y.; Gal A.; Weiner S.; Addadi L.; Fratzl P. On the Phase Diagram of Calcium Carbonate Solutions. Advanced Materials Interfaces 2017, 4 (1), 1600076 10.1002/admi.201600076.

[ref32] Avaro J. T.; Wolf S. L. P.; Hauser K.; Gebauer D. Stable Prenucleation Calcium Carbonate Clusters Define Liquid–Liquid Phase Separation. Angew. Chem., Int. Ed. 2020, 59 (15), 6155–6159. 10.1002/anie.201915350. PMC7187218 31943581

[ref33] Dobson P. S.; Bindley L. A.; Macpherson J. V.; Unwin P. R. Atomic Force Microscopy Investigation of the Mechanism of Calcite Microcrystal Growth under Kitano Conditions. Langmuir 2005, 21 (4), 1255–1260. 10.1021/la0480787. 15697268

[ref34] CRC Handbook of Chemistry and Physics; Lide D. R., Ed.; CRC Press: Boca Raton, FL, 2005.

[ref35] Bard A. J.; Faulkner L. R. Electrochemical Methods. Fundamentals and Applications; Wiley & Sons, 2001.

[ref36] Asta M. P.; Fernandez-Martinez A.; Alonso J.; Charlet L.; Findling N.; Magnin V.; Ruta B.; Sprung M.; Westermeier F. Nanoscale Ion Dynamics Control on Amorphous Calcium Carbonate Crystallization: Precise Control of Calcite Crystal Sizes. J. Phys. Chem. C 2020, 124 (46), 25645–25656. 10.1021/acs.jpcc.0c08670.

[ref37] Farhadi Khouzani M.; Chevrier D. M.; Guttlein P.; Hauser K.; Zhang P.; Hedin N.; Gebauer D. Disordered Amorphous Calcium Carbonate from Direct Precipitation. CrystEngComm 2015, 17 (26), 4842–4849. 10.1039/C5CE00720H.

[ref38] Genovese D.; Montalti M.; Otálora F.; Gómez-Morales J.; Sancho-Tomás M.; Falini G.; García-Ruiz J. M. Role of CaCO_3_° Neutral Pair in Calcium Carbonate Crystallization. Cryst. Growth Des. 2016, 16 (8), 4173–4177. 10.1021/acs.cgd.6b00276. PMC4974600 27512345

[ref39] Verch A.; Morrison I. E. G.; van de Locht R.; Kröger R. In Situ Electron Microscopy Studies of Calcium Carbonate Precipitation from Aqueous Solution with and without Organic Additives. J. Struct. Biol. 2013, 183 (2), 270–277. 10.1016/j.jsb.2013.05.017. 23742840

[ref40] Rodriguez-Navarro C.; Burgos Cara A.; Elert K.; Putnis C. V.; Ruiz-Agudo E. Direct Nanoscale Imaging Reveals the Growth of Calcite Crystals via Amorphous Nanoparticles. Cryst. Growth Des. 2016, 16 (4), 1850–1860. 10.1021/acs.cgd.5b01180.

[ref41] Lopez-Berganza J. A.; Chen S.; Espinosa-Marzal R. M. Tailoring Calcite Growth through an Amorphous Precursor in a Hydrogel Environment. Cryst. Growth Des. 2019, 19 (6), 3192–3205. 10.1021/acs.cgd.9b00062.

