# Supplementary material for: Role of Mass Transport in the Deposition, Growth, and Transformation of Calcium Carbonate on Surfaces at High Supersaturation
Source: Cryst Growth Des. 2022 Jun 27;22(8):4721–9. doi: 10.1021/acs.cgd.1c01505 (PMC9354018; doi:10.1021/acs.cgd.1c01505)
Supplement: Supplementary file 1 — cg1c01505_si_001.pdf [file cg1c01505_si_001.pdf]

Supplementary Information

Role of Mass Transport in the Deposition, Growth and Transformation of Calcium Carbonate on Surfaces at High Supersaturation

Ian J. McPherson,[a] *Massimo Peruffo,*[a,b] *and Patrick R. Unwin*[a]*

[a]Department of Chemistry, University of Warwick, Gibbet Hill Road, Coventry, CV4 7AL

[b]Present Address: Johnson Matthey Hydrogen Technologies Limited, Lydiard Fields, Great Western Way, Swindon SN5 8AT.

*p.r.unwin@warwick.ac.uk

Contents

S1. Crystal Volume Calculation S2

S2. Micro-Raman investigations S4

S3. In situ IR S6

S4. References S7

S1. Crystal Volume Calculation

The major diagonal (d_{maj}) and the minor diagonal (d_{min}) of each crystal were measured at different times during the growth and the average volume of each of the 3 chosen crystals was determined. The ratio d_{maj} / d_{min} (1.24 ±0.08) was very close to the ideal ratio of the geometry of the growing unit cell considered (Figure S1), i.e. 1.233 (see below), allowing calculation of the volume of the crystals considering the isotropic growth of an ideal crystal along the more stable {104} planes.

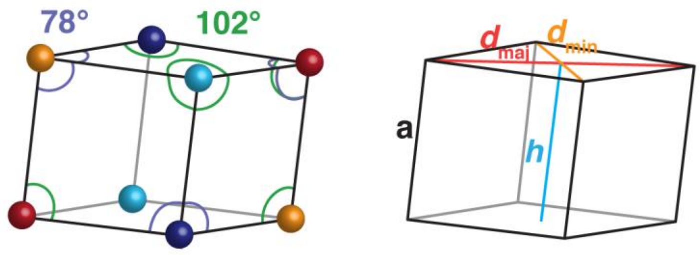

Figure S1. Representation of a rhombohedral calcite crystal with isotropic growth along the {104} planes.

Values for the edge a, d_{maj} and d_{min} were determined using crystallographic software (CrystalMaker 7.1, CrystalMaker Software Limited, UK) and were 4.65 Å, 9.980 Å and 8.095, respectively. The volume of the growing crystals was then calculated from the relation between the major diagonal (d_{maj}) and the volume of the rhombohedron. First the area of the base of the rhombohedron, A_b, can be written in terms of d_{maj} using the relation $d_{maj} = d_{min} \times 1.233$ (Eq S1).

$$A_b = \frac{d_{maj} d_{min}}{2} = \frac{d_{maj}^2}{2.466} \qquad \text{S1}$$

The height (h) of a perfect rhomb is related to the edge length, a, by Eq S2, where α (19.135°) is the angle between a and h.

$$h = a \cos \alpha \approx 0.44 d_{\mathrm{maj}} \qquad \text{S2}$$

Here, however, the height was assumed to be half that of the other lateral dimensions, given that only growth on one face contributes to the height, compared to growth on two opposite faces for the other lateral dimensions (a reasonable assumption based on experimental observations[1]) giving the formula for the rhomb volume based on its major diagonal as Eq S3.

$$V \approx 0.089 d_{\mathrm{maj}}^3 \qquad \text{S3}$$

S2. Micro-Raman investigations

Figure S2 reports a typical micro-Raman spectrum recorded on glass substrates after a calcium carbonate deposition experiment; the beam was focused on a crystal and in Table 1 are reported the peak position of the spectrum recorded and the corresponding literature values.[2,3] The comparison confirms the presence of pure calcite crystals on the surface of the glass substrate.

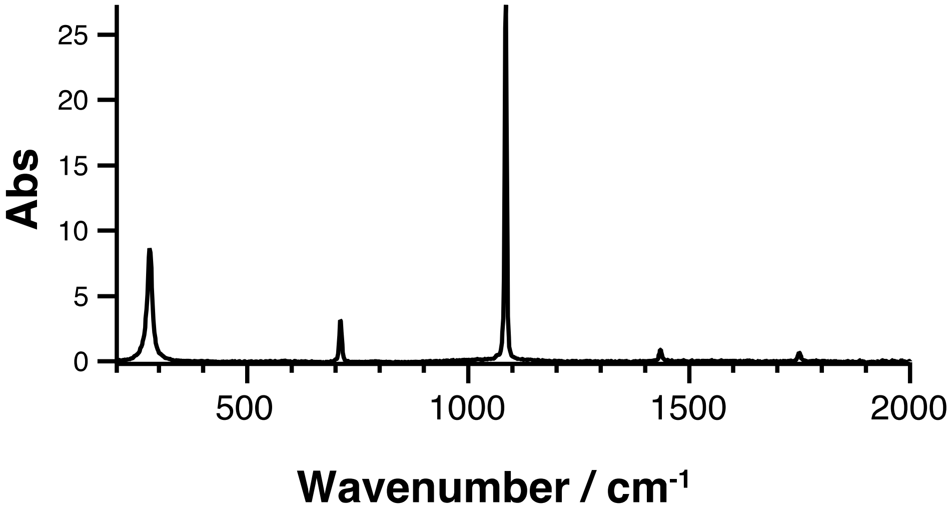

Figure S2. Micro-Raman spectrum with the laser beam focused on a calcite crystal on glass substrate after deposition within the optical cell.

Table S1 Experimental and tabulated Raman peaks for a calcite crystal.

Experimental peak (cm^{-1})	Tabulated peak (cm^{-1})	Vibration mode
280	280	Lattice mode
710	710	In plane bending
1086	1086	Symmetric stretching
1435	1435	Symmetric stretching
1749	1749	Overtones

S3. In situ IR

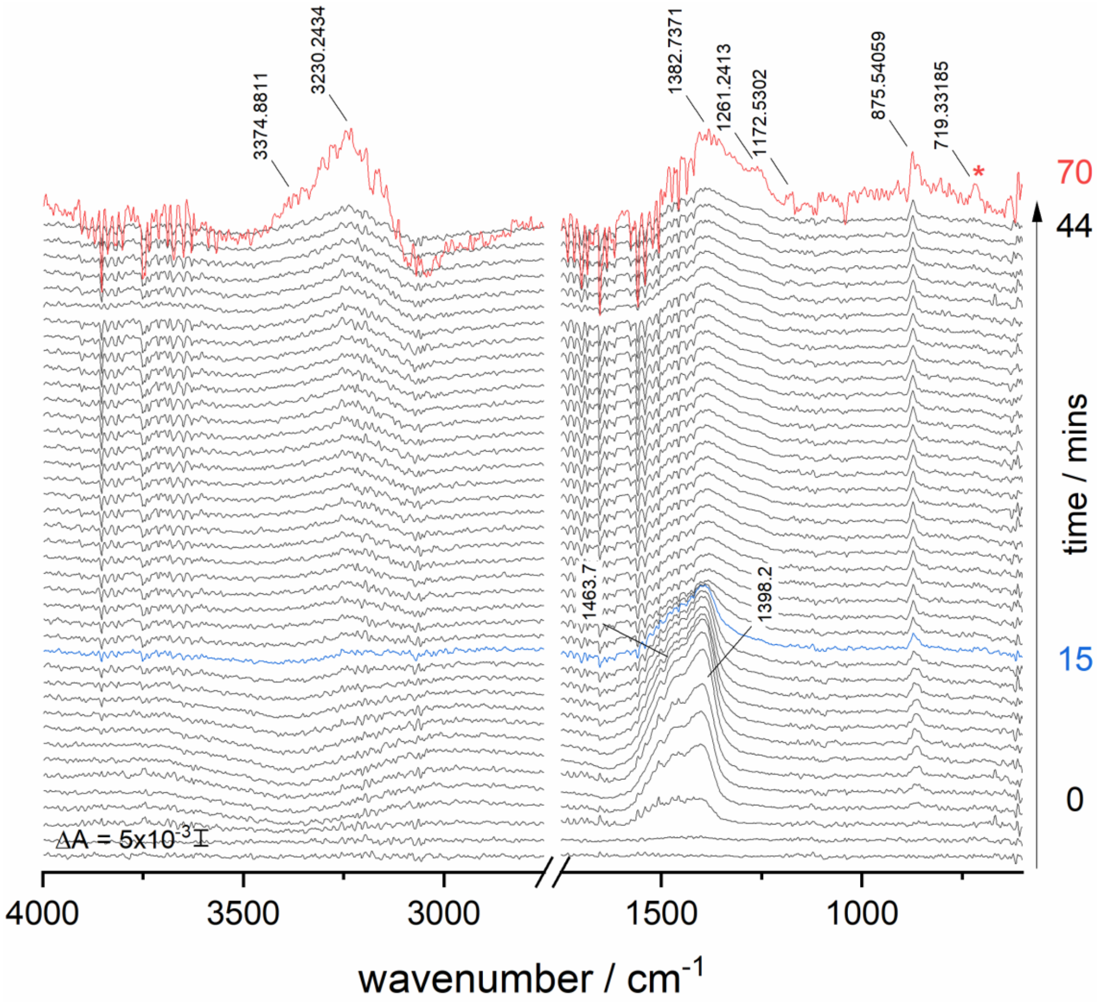

Figure S3. In situ ATR-IR spectra recorded during $CaCO_3$ deposition. * indicates the ν_4 peak close to that expected for calcite (711 cm^{-1}).[4] No spectra were recorded between 44 mins and 70 mins.

S4. References

(1) Adobes-Vidal, M.; Pearce, H.; Unwin, P. R. Tracking the Dissolution of Calcite Single Crystals in Acid Waters: A Simple Method for Measuring Fast Surface Kinetics. *Phys. Chem. Chem. Phys.* **2017**, *19* (27), 17827–17833. https://doi.org/10.1039/C7CP02252B.

(2) Rutt, H. N.; Nicola, J. H. Raman Spectra of Carbonates of Calcite Structure. *J. Phys. C: Solid State Phys.* **1974**, *7* (24), 4522–4528. https://doi.org/10.1088/0022-3719/7/24/015.

(3) Gabrielli, C.; Jaouhari, R.; Joiret, S.; Maurin, G. In Situ Raman Spectroscopy Applied to Electrochemical Scaling. Determination of the Structure of Vaterite. *Journal of Raman Spectroscopy* **2000**, *31* (6), 497–501. https://doi.org/10.1002/1097-4555(200006)31:6<497::AID-JRS563>3.0.CO;2-9.

(4) Weir, C. E.; Lippincott, E. R. Infrared Studies of Aragonite, Calcite, and Vaterite Type Structures in the Borates, Carbonates, and Nitrates. *J. Res. Natl. Bur. Stan. Sect. A.* **1961**, *65A* (3), 173. https://doi.org/10.6028/jres.065A.021.
